# Maternal dietary diversity during lactation and associated factors in Palghar district, Maharashtra, India

**DOI:** 10.1371/journal.pone.0261700

**Published:** 2021-12-29

**Authors:** Sunil Rajpal, Abhishek Kumar, Ruby Alambusha, Smriti Sharma, William Joe

**Affiliations:** 1 Department of Economics, FLAME University, Pune, India; 2 Centre for Studies in Economics and Planning, Central University of Gujarat, Gujarat, India; 3 Institute of Economic Growth, Delhi, India; 4 Nutrition, Tata Trusts, R. K. Khanna Tennis Stadium, Africa Avenue, Delhi, India; 5 Population Research Centre, Institute of Economic Growth, Delhi, India; 6 International Institute for Population Sciences, Mumbai, India; Sabzevar University of Medical Sciences, ISLAMIC REPUBLIC OF IRAN

## Abstract

Dietary adequacy and diversity during the lactation period are necessary to ensure good health and nutrition among women and children. Behavioral interventions pertaining to health and nutrition counselling during pregnancy and lactation are critical for awareness about dietary diversity. The issue assumes salience for marginalized communities because of the Covid-19 pandemic and associated economic and societal disruptions. This paper assesses the dietary patterns among 400 lactating mothers in the tribal-dominated district of Palghar in Maharashtra, India in 2020. The study is based on primary data regarding consumption of 10 food groups among women across 10 food groups based on 24-hour recall period. The primary outcome variable was binary information regarding Minimum Dietary Diversity defined as consumption from at least 5 food groups. Econometric analysis based on multilevel models and item-response theory is applied to identify food groups that were most difficult to be received by mothers during the early and late lactation period. We find that the daily diet of lactating mothers in Palghar primarily consists of grains, white roots, tubers, and pulses. In contrast, the intake of dairy, eggs, and non-vegetarian food items is much lower. Only Half of the lactating women (56.5 percent; 95% CI: 37.4; 73.8) have a minimum diversified diet (MDD). The prevalence of lactating women with MDD was higher among households with higher income (73.1 percent; 95% CI: 45.2; 89.9) than those in lower income group (50.7 percent; 95% CI: 42.3; 58.9). Lactating Women (in early phase) who received health and nutrition counseling services are more likely (OR: 2.37; 95% CI: 0.90; 6.26) to consume a diversified diet. Food groups such as fruits, meat, poultry, fish, nuts, and seeds were among the rare food items in daily diet. The dietary pattern lacking in fruits, nuts, and heme (iron) sources indicates more significant risks of micronutrient deficiencies. The findings call for improving dietary diversity among lactating mothers, particularly from the marginalized communities, and are driven by low consumption of dairy products or various fruits and vegetables. Among the different food items, the consumption of micronutrient-rich seeds and nuts is most difficult to be accessed by lactating mothers. Also, diet-centric counseling and informing lactating mothers of its benefits are necessary to increase dietary diversity for improving maternal and child nutrition.

## Introduction

Dietary intake of women is central in determining their own as well as children’s’ overall growth and development. Nutritional deficiencies via poor dietary intake results in growth faltering (including stunting), and is also associated with poor cognitive and educational outcomes among children causing adverse social and economic implications in later life. Studies have asserted that nutritious foods and diverse diet—especially during pregnancy and lactation–is critical for child’s optimal physical and mental growth throughout life [1]. Numerous studies have observed a strong bearing of mother’s dietary intake on their child’s diet and their nutritional outcomes [2, 3]. Given the high nutritional requirement during pregnancy and lactation, an adequate and a well diverse diet among women during the same is critical for optimum physical growth as well as cognitive development of children [4].

Dietary deprivations in India remains high despite rapid economic growth and notable health advancements [5]. In this regard, it is worth noting the case of Maharashtra where substantially high economic growth is accompanied by poor nutritional status among women and children. Despite being among the richest state in India, about half of the women in reproductive age in Maharashtra are unable to attain a diversified diet and are anemic [6]. Further, estimates from National Family Health Survey (NFHS hereafter) 2015–16 shows that more than half of the mothers did not receive any benefits from ICDS during pregnancy and breastfeeding [6]. Further, one in every two women did not receive any food and nutrition supplement during pregnancy and breastfeeding under Integrated Child Development Services (ICDS hereafter) program in Maharashtra. The poor state of dietary consumption among women is also accompanied by poor nutrition status among children. For instance, more than 60 percent of children (below 5 years) in Maharashtra suffer from at least one form of anthropometric failures viz. stunting or wasting or underweight [6]. In terms of global comparison, the prevalence of child underweight in Maharashtra is higher than a few underdeveloped countries such as Bangladesh, Afghanistan, and Mozambique [7, 8].

Given the increasing policy efforts under POSHAN *Abhiyaan*, it is disconcerting to observe a stagnancy in nutritional improvements in various parts of India. Against this background, the ‘Project Spotlight’ initiative was launched by Tata Trusts in selected districts of Maharashtra including Palghar to strengthen the health and nutrition services under ICDS in rural and tribal blocks. An important objective of the intervention was to improve nutritional practices and seeking behavior of the communities at individual, family and community level. Counselling on maternal diet during pregnancy and lactation period is necessary to support healthy maternal diet [9]. Postpartum period is of high relevance for motivating dietary changes because women are more likely to follow-up with advice on health and nutrition [10]. The initiative assumes further relevance as low coverage of program interventions disproportionately affects the most vulnerable and destitute sections of the state. It is crucial to take cognizance of marginalized groups particularly those from tribal communities (Scheduled Tribe, ST) as their population in absolute terms is more than 10.5 million in the state of Maharashtra. The tribal communities in India continued to suffer from economic deprivations because of several social and policy factors such as geographical accessibility, erosion of indigenous laws of the tribal communities–ensuring environmental management, conservation and preservation of the natural resource base. Further, instances like industrialization and minig operations have led to the uprooting of tribal villages leading to the loss of their traditional occupations, agricultural land, and houses. Importantly, due to an interlocking nature of tribal identities–i.e., economic and social deprivation–it is very difficult for them to take a leap out of this vicious cycle of food deprivations and therefore malnutrition. The problems are expected to have intensified due to the economic disruptions caused by the COVID-19 pandemic.

Previous studies have identified marital status, household membership status, ethnicity, participating in household decision making as some of the important factors associated with dietary diversity of women [11]. Further according to a few studies, factors such as education, age, race have positive associations with increased dietary diversity, whereas, it was negatively correlated with increased parity, single and non-working females [12]. Importantly, the likelihood of healthy food habits was higher for females from households with better occupational position and economic well-being [13]. Among children also, household wealth has strong bearing on dietary diversity in India [14]. While previous studies have identified the patterns and association between women’s dietary diversity and child’s nutrition outcomes, but evidence on underlying factors–socioeconomic and intervention related–associated with such low levels of dietary diversity has been overlooked. More importantly, there is very little evidence on the dietary intake of women during lactation–which assumes policy salience. Therefore, it is imperative to have an understanding on the levels of dietary quality of women during lactation. The estimates can offer valuable insights on important policy questions such as, which of the food groups are most difficult to be received by women, particularly during the lactation period.

This paper aims to assess the dietary diversity among lactating women in the tribal dominated (37%) district of Palghar in Maharashtra. While a few studies have previously assessed the levels of dietary intake and diversity among children [14, 15], but there is a dearth of evidence on association of nutrition education and counselling services on improving dietary intake among lactating mothers. Also, it is important to understand the dietary intake and diversity associated with early (first six months period after child birth) and late lactation period (6–24 months period after child birth) as both are associated with the window of first 1000 days that is critical for understanding rapid increase in anthropometric failure among children [16, 17]. Given that children’s’ dietary requirements in the first six months after birth is mostly based on breastfeeding (or exclusive breastfeeding), it is imperative to analyze the dietary diversity of mothers during early lactation phase in silo. Thus, we analyzed the dietary diversity for lactating mothers during the first 24 months after childbirth, which consisted early lactation period late lactation periods. The paper has four specific objectives: (a) to identify the consumption patterns of food groups across major meals of the day among lactating mothers; (b) to assess the levels of minimum dietary diversity among lactating mothers during early and late lactation phases across socioeconomic correlates; (b) to identify the food groups which are most difficult to attain or rarely consumed; (c) to test the association of health and nutrition counselling with dietary diversity. It is worth mention here that early lactation refers to the first six months of lactation to the child and late lactation refers for children aged 7 to 24 months.

It may be noted that “dietary diversity” is the most widely used indicator reflecting the micronutrient intake and quality of dietary pattern among adults as well as children [18, 19]. It is defined as the sum of well identified food groups consumed during a certain period of time. Dietary diversity is a reliable proxy for measuring nutrient intake, which simply reveals what is there in the family’s pot, instead of complex dietetics details [20, 21]. We used “Minimum Dietary Diversity” (MDD hereafter) as a primary indicator to assess the levels of dietary diversity. A woman is said to have MDD when she consumes at least five out of 10 defined food groups in previous day or night [22]. This indicator was developed by WHO primarily to assess for micronutrient adequacy–a critical dimension of diet quality.

## Methods

### Data collection

In this cross-sectional study, data was collected from 400 lactating mothers from 40 anganwadi centers (AWCs) spread across 8 administrative blocks of the Palghar district in Maharashtra, India. AWC are a part of India’s health care system and provide basic health care services. AWCs were selected based on a stratified random sample with refurbishment status of AWC used as a layer for stratification (fully refurbished AWCs and partially refurbished AWCs)

Sample size was calculated using the formula, n = *Z*^2^*p*(1-p)/d^*2*^

Where, Z = 1.96, p = Proportion of pregnant women age 15–49 years who are anaemic in Maharashtra (.49) (NFHS-4), q = 1-p and d = margin of error = 5%. Based on above formula a total of 400 mothers in in early and late lactation phases. The list of beneficiaries was taken from AWC, where more than required number of beneficiaries were available the selection of beneficiaries was done randomly and where the required number of beneficiaries were not available same type of adjacent AWC was visited for covering the sample size.

A sample of 200 lactating mothers (early phase; with children aged 0–6 months) were interviewed in 40 randomly selected AWCs in 8 blocks to understand the services they received during their antenatal period. Similarly, a sample of 200 lactating mothers (later phase, with children aged 6 to 12 months) were interviewed in 40 AWC area across 8 blocks to assess services received during six months after delivery. The list of beneficiaries and telephone details were obtained from the AWC registers and the respondents were randomly selected from the list. Due to Covid-19 and as per Government norms to maintain social distancing and travel restriction imposed, the study was conducted during May to June, 2020 through telephonic interviews by IQVIA Consulting and Information and Services Private Limited with written ethical approval from the SIGMA Institutional Review Board. A comprehensive set of tools were prepared for conducting the survey targeting diverse groups. The tools were translated into Marathi language. The consent for the same was not required as the data was analyzed anonymously. The study did not include minors.

### Outcomes and predictors

Information on 10 food group items was collected based on a 24-hour recall period: Grains, White Roots and Tubers; Pulses; Nuts and Seeds; Dairy; Meat, Poultry, Fish; Eggs; Dark green leafy vegetables; Other Vitamin A rich fruits/veg; Other vegetables and other fruits. Minimum dietary diversity (MDD) is defined as consumption from at least 5 food groups and is coded “1” if the women is consuming diversified diet and “0” otherwise [22]. The socio-economic variables for the analyses include women’s education, block level, employment status, and income of the household, age of women and ration card. For women’s education two categories were created: above and below primary with below primary being the base category. For ration card three categories were created: above poverty line, below poverty line and do not know. Three categories were created for age of women: Less than 24 years (reference category), 25 to 29 years and greater than 30 years. Income (in Indian National Rupee, INR) was categorized as: INR 0–9000 (reference category) and more than INR 9000. Income, age and education were continuous variables which were categorized. Counselling on health and nutrition was also included as a covariate. For this, dichotomous variable was constructed from the questionnaire asking “whether mothers received health and nutrition counselling from *Anganwadi* Workers (AWWs) during lactation?” (Yes = 1/No = 0). Counselling on health and nutrition by AWWs to pregnant and lactating mothers is one of the key interventions under ICDS.

### Statistical and econometric analyses

The univariate analysis describes the trend and patterns in consumption of different food items by lactating women. The meal frequency has also been analyzed on the basis of time during the day the specific food item was consumed. Five categories were created for meal frequency. To understand how each dietary item is associated with latent features of a women, we have applied item response theory framework for analysis [23–25]. Based on a likelihood ratio test for choice of parameterization, a two-parameter logistic model is used to evaluate the association between the latent trait of the women and the dietary items consumed. The model distinguishes between the items which are more commonly consumed than those which are less consumed. An item characteristic curve (ICC) is plotted to describe the probability that women consume a specific food item. The probability of consumption is a function of both the food item properties as well as the latent traits of the women. Items with large discrimination parameter thus can effectively distinguish between varying levels of latent trait.

Further, the population attributable risk (PAR) analysis framework is used to understand the potential of various food groups in increasing the MDD score in the study area [26]. Based on postestimations from logistic regression model, the potential dietary diversity (PDD) score is computed to estimate the increment in the proportion of lactating mothers with minimum dietary diversity if some of the food groups are consumed universally or are made available to all. The PDD can be interpreted as percentage increase in dietary diversity that would result because of a counterfactual scenario of cent per cent consumption of a particular food group. For example, a PDD value of 5 percent for dairy products food group indicates that universal consumption of dairy products in the population group would increase dietary diversity by 5 percentage points while keeping all other factors unchanged as observed. Following is the standard formulae for estimating PDD based on above mentioned logistic regression model:

$$PDD=\frac{(RR-1)*P_{e}}{(RR-1)*P_{e}+1}$$

Where, RR is relative risks (odds), P_e_ is the prevalence of the food group of interest and PDD is potential dietary diversity. The estimations were carried out using Stata package *regpar’* [26].

Further, the association of diversified dietary intake with the socio-economic characteristics was examined using multilevel logistic regression models with the dichotomized diversified diet indicator (Yes = 1 and No = 0) as the dependent variable and other socio-economic variables as the key explanatory variables. The multilevel analysis uses the two-level hierarchical structure of data with diet information on women (level-1) being nested within administrative blocks (level-2) for regression. We also estimated the variance partition coefficient to understand between-block variations in dietary diversity [27]. The whole analysis was conducted separately for lactating mothers to children aged 0–6 months (early lactation) and lactating mothers to children aged 6–24 months (late lactation). All the analysis is performed using statistical software Stata 15.0.

## Results

Among mothers in early lactation phase (first six months after child birth), 56.2 percent were less than 24 years of age, 34 percent were between the age of 25–29 years and 9.7 percent were above 30 years or above (S1 Table). 61 percent had up to primary-level education, and rest 39 percent were educated above primary. In terms of household income, 26 percent of lactating mothers of children aged 0–6 months belonged to household income more than INR 9000 (1 USD ~ INR 70). 13.5 percent of the interviewed mothers were employed. Out of the total sample for mothers in late lactation phase (children aged 6–24 months), 55.5 and 36.0 percent were 0 to 24 years and 25 to 29 years old, respectively. Approximately 80 per cent of the lactating women (7–24 months) belonged to households with income less than INR 9000 per month.

Three-fourth of mothers during early lactation (74.5 percent) and 66.5 percent during late lactation did not have fruits even single time in last 24 hours (Table 1). The consumption of dairy products and eggs was also low as about half of all lactating women did not have a single meal including dairy products and eggs. On the other hand, the grains and pulses-based diets were reported to be most frequent in daily diet of lactating women in both early as well as late lactation period. For example, about 42 percent and 37.5 percent of early and late lactating women respectively, were reported to have meals including grains, white roots and tubers 4 times in a day. The consumption of non-vegetarian diet was also not included in the daily meals of lactating women. About 69 percent and 76 percent of women during early and late lactation respectively did not have meat, poultry, or fish in their daily diets. The consumption of green leafy vegetables was observed to be present (i.e., at least 1 time in a day) among only 29 percent of mothers during early lactation and 35 percent among those during late lactation.

**Table 1:** Distribution of meal frequency by food groups among lactating mothers in Palghar, 2018.

Meal frequency:	0		1		2		3		4		Total	
Early phase	N	%	N	%	N	%	N	%	N	%	N	%
Grains, White Roots and Tubers	17	8.5	37	18.5	45	22.5	17	8.5	84	42	200	100
Pulses	38	19	42	21	46	23	20	10	54	27	200	100
Nuts and Seeds	176	88	18	9	5	2.5	1	0.5	0	0	200	100
Dairy	111	55.5	81	40.5	4	2	4	2	0	0	200	100
Meat, Poultry, Fish	138	69	48	24	8	4	5	2.5	1	0.5	200	100
Eggs	101	50.5	86	43	12	6	1	0.5	0	0	200	100
Dark green leafy vegetables	90	45	58	29	40	20	11	5.5	1	0.5	200	100
Other Vitamin A rich fruits/veg	141	70.5	31	15.5	27	13.5	1	0.5	0	0	200	100
Other vegetables	71	35.5	78	39	35	17.5	15	7.5	1	0.5	200	100
Other fruits	149	74.5	42	21	7	3.5	2	1	0	0	200	100
**Late phase**												
Grains, White Roots and Tubers	16	8	31	15.5	46	23	32	16	75	37.5	200	100
Pulses	30	15	49	24.5	40	20	39	19.5	42	21	200	100
Nuts and Seeds	167	83.5	25	12.5	8	4	0	0	0	0	200	100
Dairy	100	50	85	42.5	11	5.5	2	1	2	1	200	100
Meat, Poultry, Fish	152	76	37	18.5	9	4.5	2	1	0	0	200	100
Eggs	116	58	76	38	8	4	0	0	0	0	200	100
Dark green leafy vegetables	73	36.5	70	35	51	25.5	6	3	0	0	200	100
Other Vitamin A rich fruits/veg	146	73	29	14.5	25	12.5	0	0	0	0	200	100
Other vegetables	99	49.5	56	28	31	15.5	14	7	0	0	200	100
Other fruits	133	66.5	59	29.5	7	3.5	1	0.5	0	0	200	100

Overall, about 56.5 percent of mother in their early lactation period and 52.5 percent of mothers in their late lactation period had minimum dietary diversity (Table 2). Across educational groups, no significant difference in the prevalence of minimum dietary diversity was observed. However, a clear gradient in the prevalence of MDD was observed across income groups with relatively higher diversity among economically affluent households. While 51 percent (95% CI: 42.3, 58.9) of lactating mothers (early lactation) from lowest income group had MDD, it was more than double (73.1 percent; 95% CI: 45.2; 89.9) among richest household group.

**Table 2:** Percentage of women with minimum dietary diversity among lactating mothers by background characteristics, Palghar, 2020.

	Early phase			Later phase		
	N	%	95% CI	N	%	95% CI
Education level						
Up to Primary	74	60.7	[36.0,80.9]	64	57.7	[31.2,80.4]
Above Primary	39	50.0	[30.9,69.1]	41	46.1	[28.9,64.3]
Below Poverty Line						
APL	8	44.4	[12.5,81.7]	10	43.5	[24.1,65.1]
BPL	99	58.9	[39.9,75.6]	86	57.0	[41.8,70.9]
DNK	6	42.9	[8.2,86.3]	09	34.6	[07.5,77.5]
Employment						
Not employed	100	57.8	[38.5,75.0]	89	52.0	[32.1,71.4]
Employed	13	48.1	[25.8,71.3]	16	55.2	[33.0,75.5]
Income (Rs.)						
0–9000	75	50.7	[42.3,58.9]	78	49.7	[41.6,57.8]
More than 9000	38	73.1	[45.2,89.9]	27	62.8	[45.3,77.5]
Block ID						
Dahanu	4	25.0	[25.0,25.0]	2	09.1	[9.1,9.1]
Jawahar	32	80.0	[80.0,80.0]	23	65.7	[65.7,65.7]
Mokhada	20	60.6	[60.6,60.6]	28	66.7	[66.7,66.7]
Palghar	20	90.9	[90.9,90.9]	12	80.0	[80.0,80.0]
Talasari	6	46.2	[46.2,46.2]	2	20.0	[20.0,20.4]
Vada	6	40.0	[40.0,40.0]	12	63.2	[63.2,63.3]
Vasai	1	8.3	[08.3,08.3]	04	23.5	[23.3,23.7]
Vikramgad	24	49.0	[49.0,49.2]	22	55.0	[54.9,55.3]
Mother’s Age						
Less than 24 years	46	44.2	[23.2,67.6]	57	51.4	[34.8,67.6]
25 to 9 years	40	63.5	[40.9,81.4]	37	51.4	[33.1,69.3]
More than 30 years	15	83.3	[47.7,96.5]	11	64.7	[17.8,93.9]
Overall	113	56.5	[37.4,73.8]	105	52.5	[34.9,69.5]

N = Total Number of Observations.

A similar gradient was noted among mothers in their late lactation period as well with 49.7 percent (95% CI: 41.6, 57.8) and 62.8 percent (95% CI: 45.3; 77.5) MDD reported in poorest and richest groups, respectively (Table 2). Further, dietary diversity was reported to be higher among mothers from older age groups compared to the younger ones. For instance, the prevalence of MDD among lactating mothers aged 30 years or above was 83.3 percent (95% CI: 47.7; 96.5) and 64.7 percent (95% CI: 17.8; 93.9) during early and late lactation respectively. On the other hand, it was 44.2 percent (95% CI: 23.2; 67.6) and 34.8 percent (95% CI: 34.8; 67.6) among lactating women from younger age groups.

**Fig 1:**
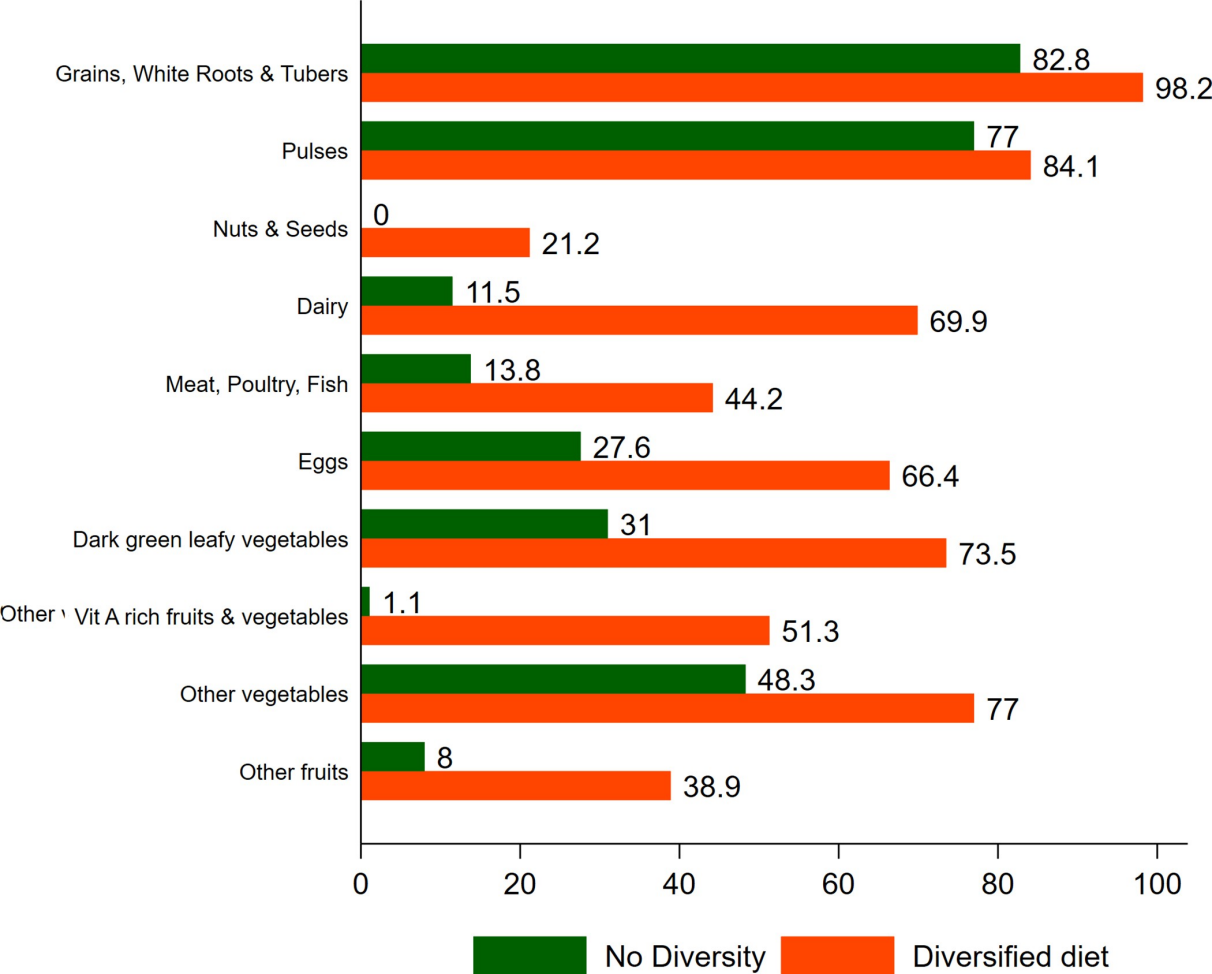
Consumption of food items by dietary diversity groups among lactating mothers (Early phase), Palghar, 2020. Blue Ref: No diversity Orange Ref: Diversified diet.

**Fig 2:**
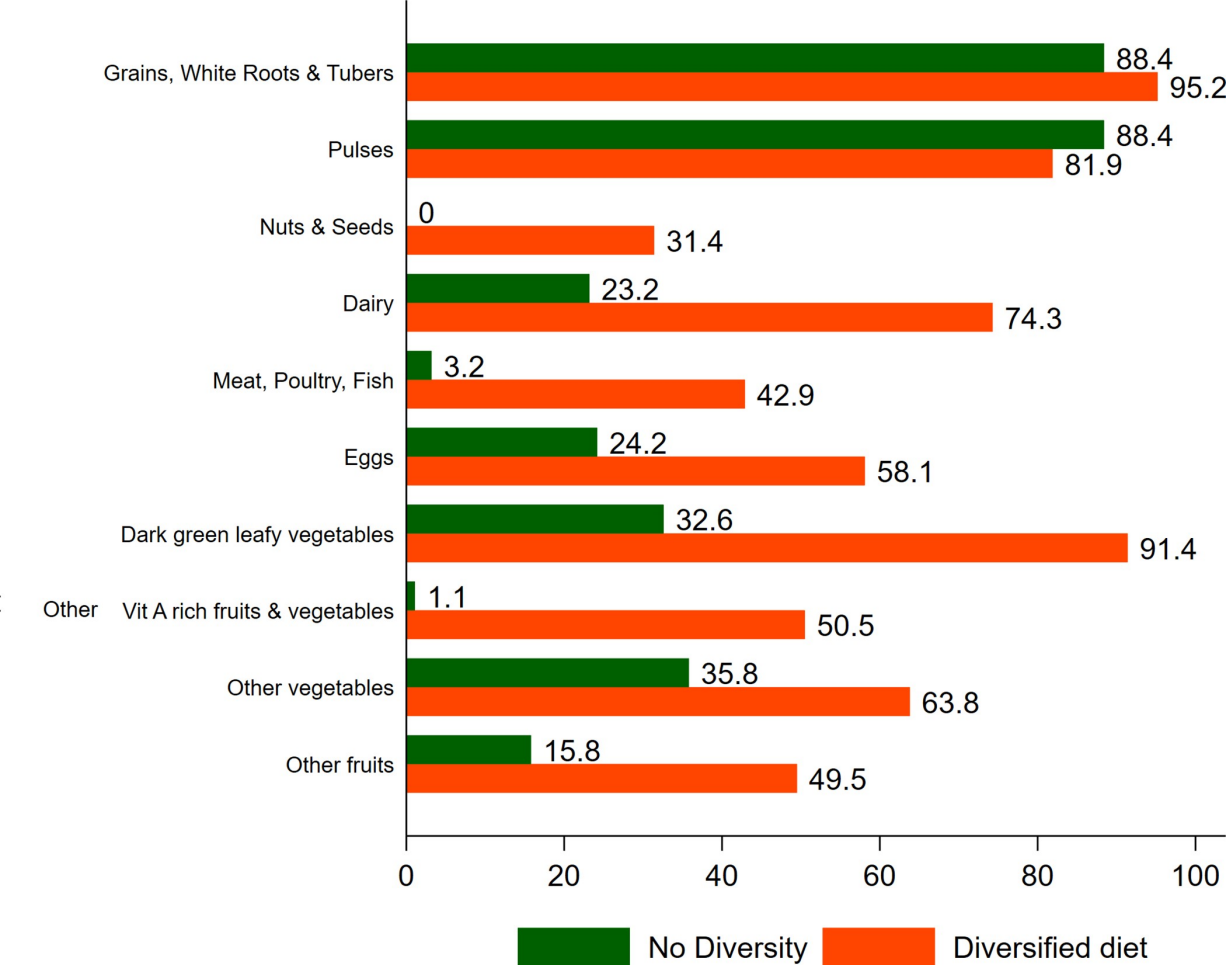
Consumption of food items by dietary diversity groups among lactating mothers (Early phase), Palghar, 2020. Blue Ref: No diversity Orange Ref: Diversified diet.

Figs 1 and 2 presents the patterns in consumption of specific food items among mothers based on dietary diversity during early and late lactation respectively. Intake of each food item is higher among women who are consuming a diversified diet. Substantial differences were observed between the two groups (i.e., with and without MDD) with respect to consumption of Nuts and Seeds; Dairy; Meat, Poultry, Fish; Eggs; Dark green leafy vegetables; Other Vit A rich fruits and vegetables; Other vegetables and other fruits. Among mothers with no dietary diversity, only the consumption of grains, white roots and tubers is high (82.8 percent during early lactation) and for all the rest of the groups, the consumption is subpar.

Table 3 presents the odds of attaining MDD among lactating women across socioeconomic groups. The odds of attaining MDD among lactating women (early phase) is significantly higher for richest section (OR: 3.54; 95% CI: 1.46, 8.59) compared to poorest group. In case of age groups as well, compared to younger age-group (less than 24 years), lactating women in early phase above 30 years of age are about five times (OR: 5.81; 95% CI: 1.38, 24.40) more likely to receive a minimum diversified diet. Early Lactating mothers who have received Health and Nutrition Counselling (HNC hereafter) are about 2 times more likely to consume a diversified diet.

**Table 3:** Odds ratios based on multilevel logistic regression estimates regarding association between MDD among lactating mothers and socioeconomic correlates, Palghar, 2020.

	Early phase	Later phase	All
Health & Nutrition counselling			
No^¶^	1.00	1.00	1.00
Yes	2.37	1.13	1.42
	[0.90,6.26]	[0.59,2.20]	[0.84,2.40]
Mother’s Education			
Up to Primary^¶^	1.00	1.00	1.00
Above Primary	0.59	0.51*	0.53**
	[0.28,1.21]	[0.26,0.99]	[0.33,0.85]
Employment			
Not employed^¶^	1.00	1.00	1.00
Employed	0.69	0.96	0.78
	[0.25,1.88]	[0.38,2.43]	[0.41,1.52]
Household Income (Rs.)			
0–9000^¶^	1.00	1.00	1.00
More than 9000	3.54**	1.44	2.20**
	[1.46,8.59]	[0.63,3.28]	[1.22,3.94]
Mother’s Age (Years)			
24 or Less than 24^¶^	1.00	1.00	1.00
25–29	2.46*	1.18	1.77*
	[1.15,5.27]	[0.58,2.39]	[1.06,2.95]
30 or above	5.81*	1.75	3.06*
	[1.38,24.40]	[0.46,6.62]	[1.18,7.92]
Woman’s Questionnaire			
Early Lactation (0–6 months) ^¶^			1.00
Late Lactation Period (7–24 months)			1.24
			[0.75,2.06]
N	178	189	367

^¶^ denotes reference category. The models include an intercept term.

Note: *p < .*05*.

**p < .01.

***p < .*001*. 95% CI in [].

In Table 4 the interaction effect of counselling and income are considered. It seems that women who belong to the richer group are more likely to benefit from HNC counselling. Compared to their peers who did not receive the counselling, the odds of consuming a diversified diet was about 7 times and 2 times higher for women who received HNC during early and later lactation phase respectively. Importantly, lactating mothers (during early lactation) who have received HNC training are more likely (OR: 1.24; 95% CI: 0.75, 2.06) to consume a diversified diet. Similar association was observed between HNC and dietary diversity is slightly weak in case of lactating women (OR: 1.21; 95% CI: 0.64, 2.30). The association between HNC counselling and dietary diversity among lactating mothers was more robust in a univariate framework (S2 Table).

**Table 4:** Odds ratios based on multilevel logistic regression estimates regarding association of MDD among lactating mothers with Household’s Income and nutrition counselling, Palghar, 2020.

	Early phase	Later phase	All
Health and Nutrition counselling			
0–9000*No counselling®	1.00	1.00	1.00
	[1.00,1.00]	[1.00,1.00]	[1.00,1.00]
More than 9000*No counselling	0.79	1.03	1.00
	[0.14,4.54]	[0.32,3.31]	[0.39,2.53]
0–9000*Counselled	1.21	1.00	1.06
	[0.39,3.76]	[0.48,2.08]	[0.59,1.92]
More than 9000*Counselled	7.41**	1.98	3.88**
	[1.75,31.33]	[0.62,6.27]	[1.64,9.15]
N	178	189	367

® denotes reference category. The models include an intercept term.

Note: *p < .*05*.

**p < .01.

***p < .*001*. 95% CI in [].

Estimates from Item Response Theory (IRT) based single parameter logistic regression models highlights the role of latent traits critical for consuming various food groups during lactation (Table 5). Across all 10 food groups, meal based on grains, white roots and tubers is least difficult to attain, whereas consuming nuts and seeds were found to be most difficult (Table 5). In simple words, receiving a diet rich in nuts and seeds requires most efforts by lactating (both during early and later phase) women and their households. Food groups such as meat, poultry and fish, Vitamin-A rich and other fruits have high and statistically significant discriminatory parameters of 1.19 (95% CI: 0.90; 1.49); 1.15 (95% CI: 0.85; 1.44) and 1.07 (95% CI: 0.78; 1.36) respectively. However, discrimination parameters for pulses (-1.94; 95% CI: -2.31; -1.57) and dark green leafy vegetables (-0.48; 95% CI: -0.73; -0.23) are significantly negative. The estimates for full model are presented in S3 Table. These estimates show that it is difficult to find a protein-rich diet based on meats, poultry and fish and relatively easier to observe pulses and vegetable-based meals among lactating women. Similar pattern can be observed from ICC plots as well (S1 Fig).

**Table 5:** Item difficulty parameters based on one-parameter logistic model for consumption of food groups among lactating mothers, Palghar 2020.

	Item Difficulty Parameter	
	Coefficient	95% CI
Grains, White Roots and Tubers	‒2.88***	[‒3.39 –‒2.37]
Pulses	‒1.94***	[‒2.31 –‒1.57]
Dark green leafy vegetables	‒0.48***	[‒0.73 –‒0.23]
Other vegetables	‒0.39***	[‒0.64 –‒0.14]
Dairy	00.13	[‒0.12–0.37]
Eggs	00.20	[‒0.05–0.45]
Other fruits	1.07***	[0.78–1.36]
Other Vitamin A rich fruits/veg.	1.15***	[0.85–1.44]
Meat, Poultry, Fish	1.19***	[0.90–1.49]
Nuts and Seeds	2.19***	[1.79–2.60]

Finally, Table 6 shows the potential dietary diversity achievable if all lactating mothers consume the various food items from different groups. The attribution analysis based on the PAR framework shows that provisioning of dairy product as well as meat, poultry and fish in daily intake among mothers in early lactation phase can increase the MDD score from 55% to 72%. The inclusion of these products in daily diets has highest potential attribution factor of 17%. Inclusion of diversified group of fruits, nuts and vegetables in daily diets can also increase the dietary diversity by 8% to 11%. A similar pattern is noted in case of lactating mothers in the later phases. In particular, inclusion of dairy products or meat-based food items can increase the dietary diversity significantly. Eggs can enhance dietary diversity by 7% to 8% among lactating mothers.

**Table 6:** Potential dietary diversity (%) achievable among lactating mothers through planning a universal coverage of various food group items, Palghar 2020.

Food group items	Early phase			Late phase		
	MDD	PDD	Attribution	MDD	PDD	Attribution
Grains, White Roots and Tubers	54.5	58.1	3.6	52.9	54.9	2.0
Pulses	54.5	60.5	6.0	52.9	54.9	2.0
Dairy	54.5	71.4	16.9	52.9	68.1	15.2
Meat, Poultry, Fish	54.5	71.8	17.3	52.9	82.9	30.0
Eggs	54.5	61.4	6.9	52.9	60.8	7.9
Dark green leafy vegetables, Nuts, Vita-a fruits/veg and Other fruits	54.5	62.2	7.7	52.9	64.2	11.3
Other vegetables	54.5	65.3	10.9	52.9	64.1	11.2

## Discussion

This study assessed the dietary patterns among lactating mothers in Palghar district of Maharashtra with a particular focus on examining dietary diversity. Following are the five salient findings from the present analysis: first, daily diet of most of the lactating women in Palghar contains a substantially higher component (frequency) of grains, white roots, tubers, and pulses and much lower frequency of dairy, eggs, and non-vegetarian food. In fact, one in every two lactating mothers did not have any dairy-based food (or dairy products) in even a single meal of the day. Second, about half of the lactating mothers did not have a minimum diversified diet and were confined to selected food groups in all the meals of a day. Third, a clear gradient was observed in the proportion of women with MDD across income groups with higher likelihood of diversity among women from richer households. Such patterns were further confirmed through multilevel regression models. Fourth, food groups such as fruits, meat, poultry, fish, nuts and seeds are among the most difficult components to be attained and therefore attention on these items is necessary at the policy front. Also, increase in consumption of these food groups can enhance the dietary diversity among lactating mothers. Fifth, a significant association between health and nutrition counselling and consumption of a diversified diet is observed. Estimates from intersectional model further confirms that mothers from well to do household and have received HNC are more likely to have a diversified diet.

Our findings regarding prevalence and socioeconomic patterning of MDD among lactating mothers is consistent with other studies based on nationally representative surveys (such as National Family Health Survey, NFHS) and other data sources [28]. The observed gradient in dietary diversity across income groups clearly implies the significance of household contextual and income related factors in determining dietary intake of women. In this regard, a few studies have also suggested that household dietary diversity is a potent tool for assessing household’s food insecurity and deprivation [28, 29]. Our findings regarding lack of dietary diversity among half of the lactating women calls for urgent policy attention as this huge proportion are at highest risk of micronutrient deficiencies. In this regard, it is worth noting that universal provisioning of dairy products has greater scope and potential to enhance dietary diversity score than food items such as eggs, fruits, vegetables and nuts.

We observed that meals based on grains, white roots and tubers were most frequent (as many as 4 times) in the daily dietary composition of women and more than half of sample women did not have any dairy-based food even in one meal. Such low variations in frequency may cause micronutrient deficiencies ultimately translating into maternal and child undernutrition. Studies in this regard have observed substantially high micronutrient deficiencies among women in India [30, 31]. The observed lack of diversity in daily meal composition also iterates the need for behavioral interventions to promote awareness regarding importance of including protein- and vitamin-rich food such as diary-products, fruits, and eggs [32]. A recent study based on NFHS 2015–16 also asserts that consumption of pumpkin, carrots, meat-based food, legumes and nuts among women was significantly associated with maternal education and not poverty status [14]. It is also imperative for policymakers to take cognizance of such findings and diversify the food supplementation–such as Hot Cooked Meals and Take-Home Ration provided by AWCs—at policy level. Improving dietary interventions through intervention programs assumes much salience for two underlying reasons: First, food items like pumpkins, carrots and green leafy vegetables are relatively cheaper and; second, studies observe a high concordance between service utilization by mothers during pregnancy and children thereby enhancing chances of dietary diversity [33].

Across all food groups, meals including nuts, seeds, vitamin-A rich and other fruits were most difficult to receive. In this regard, a recent study assessing nutrition status of children in Palghar district of Maharashtra having similar observations highlighted that meals were mainly composed on *dal* and rice, and almost zero component of fruits, milk and milk products, flesh food, fish, and eggs [34]. This is all the more worrisome as the district is dominated by tribal population who are already grappling with socioeconomic constraints. Such low variations in family’s diet reflects food insecurity in tribal-dominated districts can be attributed to diminishing dependence on forest livelihood and increasing agrarian crisis in the state [35]. Besides social and economic vulnerability, systematic issues like accessibility to public distribution system, low coverage of nutrition programmes aggravate the problem leading to an inter-generation cycle of malnutrition [36].

At this point, it is important to mention the limitations of study. The variable for MDD among lactating mothers is constructed from question regarding food frequency based on 24-hour dietary recall. Accordingly, the aim in this paper is to examine the number of food groups from which consumption is done on the daily basis and not to analyze the meal composition. In addition to this, the cross-sectional nature of data restricts us to infer about any causality between socioeconomic factors and maternal dietary diversity. Therefore, the conclusions made in this paper are restricted to infer about the association between outcome and explanatory variables. Importantly, the information on dietary intake of lactating women is based on telephonic survey which makes it difficult for an individual to report accurately about food consumption and hence there is a possibility of underestimation [37].

## Conclusion

In concluding, the study asserts that inadequate dietary diversity is a major concern among lactating mothers and is driven by low consumption of dairy products or various fruits and vegetables. Among the various food items, the consumption of micronutrient rich seeds and nuts is most difficult to be accessed by lactating mothers. Also, diet-centric counselling and informing lactating mothers of its benefits is necessary to increase dietary diversity for improving maternal and child nutrition. However, counselling services among poor should be supported with direct supplementation support. Supplementary nutrition programs through various central and state government initiatives such as ICDS can consider diversifying the existing cereal centric approach to a more diversified strategy by including food groups such as dairy products, eggs or fruits and nuts.

## Supporting information

S1 Fig Item characteristic curve (ICC) for consumption of food groups among lactating mothers, Palghar 2020. (DOCX)

S1 Table Sample distribution of lactating mothers by background characteristics, Palghar, 2020. (DOCX)

S2 Table Odds ratios based on multilevel logistic regression estimates regarding association between MDD among lactating mothers and nutrition counselling, Palghar, 2020. (DOCX)

S3 Table Odds ratios based on multilevel logistic regression estimates regarding association between MDD among lactating mothers and socioeconomic correlates, Palghar, 2020. (DOCX)
