# Supplementary material for: Maternal dietary diversity during lactation and associated factors in Palghar district, Maharashtra, India
Source: PLoS One. 2021 Dec 29;16(12):e0261700. doi: 10.1371/journal.pone.0261700 (PMC8716033; doi:10.1371/journal.pone.0261700)

**S1 Fig. Item characteristic curve (ICC) for consumption of food groups among lactating mothers, Palghar 2020.**


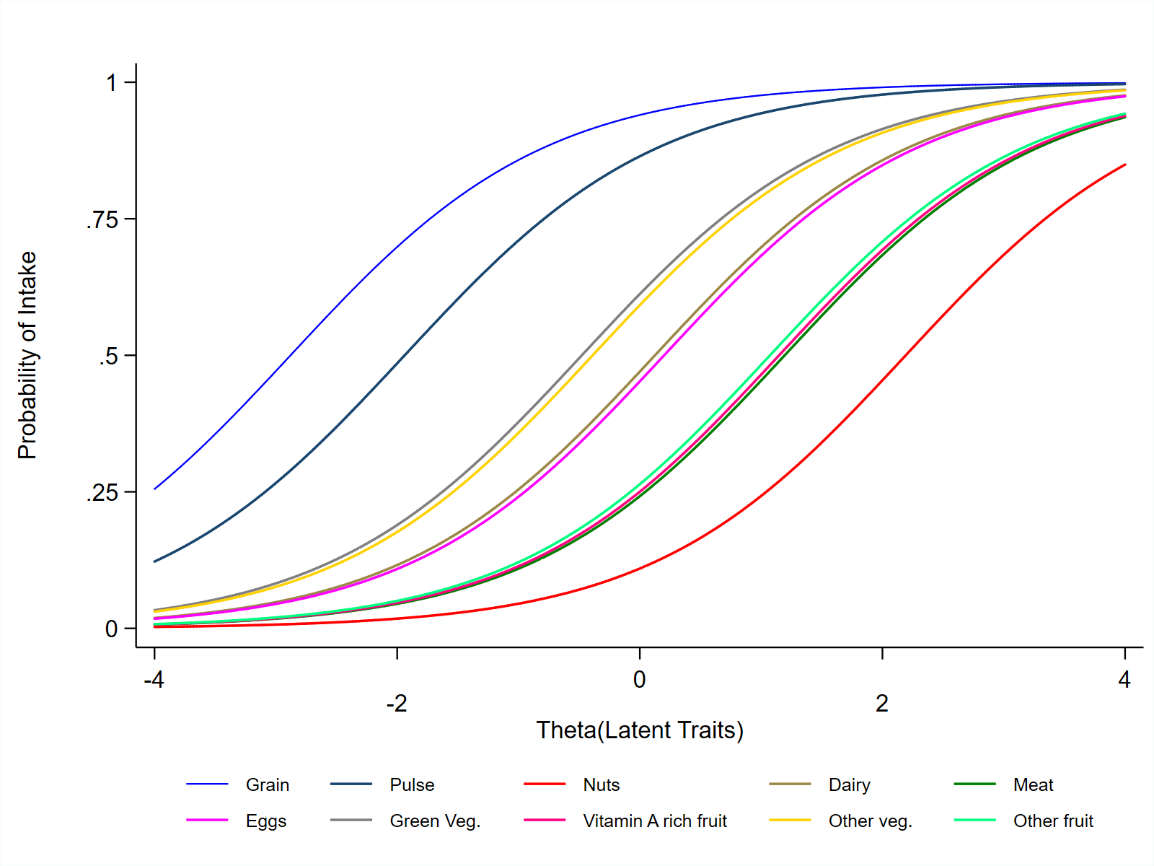

Supplement: S1 Fig — (DOCX) [file pone.0261700.s001.docx]
