# Supplementary material for: Maternal dietary diversity during lactation and associated factors in Palghar district, Maharashtra, India
Source: PLoS One. 2021 Dec 29;16(12):e0261700. doi: 10.1371/journal.pone.0261700 (PMC8716033; doi:10.1371/journal.pone.0261700)
Supplement: S1 Table — (DOCX) [file pone.0261700.s002.docx]

**S1 Table. Sample distribution of lactating mothers by background characteristics, Palghar, 2020.**

|  | Early Lactation | | Late Lactation | |
| --- | --- | --- | --- | --- |
|  | N | % | N | % |
| Education |  |  |  |  |
| Up to Primary | 122 | 61.0 | 111 | 55.5 |
| Above Primary | 78 | 39.0 | 89 | 44.5 |
| Employed |  |  |  |  |
| Not employed | 173 | 86.5 | 171 | 85.5 |
| Employed | 27 | 13.5 | 29 | 14.5 |
| Income |  |  |  |  |
| 0-9000 | 148 | 74 | 157 | 78.5 |
| More than 9000 | 52 | 26.0 | 43 | 21.5 |
| Age of woman |  |  |  |  |
| Less than 24 years | 104 | 56.2 | 111 | 55.5 |
| 25 to 9 years | 63 | 34.1 | 72 | 36.0 |
| More than 30 years | 18 | 9.7 | 17 | 8.5 |
| Block ID |  |  |  |  |
| Dahanu | 16 | 8.0 | 22 | 11.0 |
| Jawahar | 40 | 20.0 | 35 | 17.5 |
| Mokhada | 33 | 16.5 | 42 | 21.0 |
| Palghar | 22 | 11.0 | 15 | 7.5 |
| Talasari | 13 | 6.5 | 10 | 5.0 |
| Vada | 15 | 7.5 | 19 | 9.5 |
| Vasai | 12 | 6.0 | 17 | 8.5 |
| Vikramgad | 49 | 24.5 | 40 | 20.0 |
