# Supplementary material for: Maternal dietary diversity during lactation and associated factors in Palghar district, Maharashtra, India
Source: PLoS One. 2021 Dec 29;16(12):e0261700. doi: 10.1371/journal.pone.0261700 (PMC8716033; doi:10.1371/journal.pone.0261700)
Supplement: S2 Table — (DOCX) [file pone.0261700.s003.docx]

**S2 Table. Odds ratios based on multilevel logistic regression estimates regarding association between MDD among lactating mothers and nutrition counselling, Palghar, 2020.**

|  | Early Lactation | | Late Lactation | | All | |
| --- | --- | --- | --- | --- | --- | --- |
| Health and nutrition counselling | |  | |  | |  |
| No® | 1.00 | | 1.00 | | 1.00 | |
|  | [1.00,1.00] | | [1.00,1.00] | | [1.00,1.00] | |
| Yes | 1.59 | | 1.21 | | 1.35 | |
|  | [0.70,3.62] | | [0.64,2.30] | | [0.84,2.15] | |
| N | 193 | | 189 | | 382 | |

® denotes reference category. The models include an intercept term. Note: **p < .05. **p < .01. ***p < .001.* 95% CI in [ ].
