# Supplementary material for: Maternal dietary diversity during lactation and associated factors in Palghar district, Maharashtra, India
Source: PLoS One. 2021 Dec 29;16(12):e0261700. doi: 10.1371/journal.pone.0261700 (PMC8716033; doi:10.1371/journal.pone.0261700)
Supplement: S3 Table — (DOCX) [file pone.0261700.s004.docx]

**S3 Table. Odds ratios based on multilevel logistic regression estimates regarding association between MDD among lactating mothers and socioeconomic correlates, Palghar, 2020.**

|  | Early Lactation | Late Lactation | All |
| --- | --- | --- | --- |
| Health and nutrition counselling*Income |  |  |  |
| 0-9000*No counselling® | 1.00 | 1.00 | 1.00 |
|  | [1.00,1.00] | [1.00,1.00] | [1.00,1.00] |
| More than 9000*No counselling | 0.79 | 1.03 | 1 |
|  | [0.14,4.54] | [0.32,3.31] | [0.39,2.53] |
| 0-9000*Counselled | 1.21 | 1 | 1.06 |
|  | [0.39,3.76] | [0.48,2.08] | [0.59,1.92] |
| More than 9000*Counselled | 7.41** | 1.98 | 3.88** |
|  | [1.75,31.33] | [0.62,6.27] | [1.64,9.15] |
| Education |  |  |  |
| Up to Primary® | 1.00 | 1.00 | 1.00 |
|  | [1.00,1.00] | [1.00,1.00] | [1.00,1.00] |
| Above Primary | 0.5 | 0.51 | 0.51** |
|  | [0.23,1.06] | [0.26,1.00] | [0.31,0.83] |
| Employed |  |  |  |
| Not employed® | 1.00 | 1.00 | 1.00 |
|  | [1.00,1.00] | [1.00,1.00] | [1.00,1.00] |
| Employed | 0.68 | 0.97 | 0.75 |
|  | [0.24,1.88] | [0.38,2.45] | [0.39,1.45] |
| Mother’s Age |  |  |  |
| Less than 24 years® | 1.00 | 1.00 | 1.00 |
|  | [1.00,1.00] | [1.00,1.00] | [1.00,1.00] |
| 25-29 years | 2.58* | 1.17 | 1.80* |
|  | [1.19,5.56] | [0.57,2.38] | [1.08,3.01] |
| More than 30 years | 7.13* | 1.75 | 3.26* |
|  | [1.58,32.28] | [0.46,6.69] | [1.24,8.62] |
| Woman questionnaire |  |  |  |
| Pregnant women® |  |  | 1.00 |
|  |  |  | [1.00,1.00] |
| Lactating women |  |  | 1.21 |
|  |  |  | [0.73,2.01] |
| N | 178 | 189 | 367 |

® denotes reference category. The models include an intercept term. Note: **p < .05. **p < .01. ***p < .001.* 95% CI in [ ].
